# A predictive model for early diagnosis of keratoconus

**DOI:** 10.1186/s12886-020-01531-9

**Published:** 2020-07-02

**Authors:** Gracia Castro-Luna, Antonio Pérez-Rueda

**Affiliations:** 1 Department of Nursing, Physiotherapy and Medicine, The University of Almería, Almería, Spain; 2 UGC Ophthalmology, Torrecárdenas University Hospital, Almería, Spain

**Keywords:** Keratoconus, Corneal topography, High order aberrations, Coma

## Abstract

**Background:**

The diagnosis of keratoconus in the early stages of the disease is necessary to initiate an early treatment of keratoconus. Furthermore, to avoid possible refractive surgery that could produce ectasias. This study aims to describe the topographic, pachymetric and aberrometry characteristics in patients with keratoconus, subclinical keratoconus and normal corneas. Additionally to propose a diagnostic model of subclinical keratoconus based in binary logistic regression models.

**Methods:**

The design was a cross-sectional study. It included 205 eyes from 205 patients distributed in 82 normal corneas, 40 early-stage keratoconus and 83 established keratoconus. The rotary Scheimpflug camera (Pentacam® type) analyzed the topographic, pachymetric and aberrometry variables. It performed a descriptive and bivariate analysis of the recorded data. A diagnostic and predictive model of early-stage keratoconus was calculated with the statistically significant variables.

**Results:**

Statistically significant differences were observed when comparing normal corneas with early-stage keratoconus/ in variables of the vertical asymmetry to 90° and the central corneal thickness. The binary logistic regression model included the minimal corneal thickness, the anterior coma to 90° and posterior coma to 90°. The model properly diagnosed 92% of cases with a sensitivity of 97.59%, specificity 98.78%, accuracy 98.18% and precision 98.78%.

**Conclusions:**

The differential diagnosis between normal cases and subclinical keratoconus depends on the mínimum corneal thickness, the anterior coma to 90° and the posterior coma to 90°.

## Background

Keratoconus is an asymmetrical bilateral eye disease [1] in which corneal thinning and protrusion occurs in the form of a generally lower temporal cone. This corneal deformation produces a significant decrease in visual quality.

It usually appears in adolescence, progressing into the third or fourth decade [1]. Although of unknown etiology, it has been related to genetic factors [2] such as environmental factors [3, 4].

The incidence and prevalence of keratoconus are very variable. It has been seen that in Europe, the frequency would be between 5 and 23 per 100,000 people/year, and the average prevalence would be 54 per 100,000 [5]. Recently Bak-Nielsen et al. [6] have published an average incidence rate of 3.60 per 100,000 person-years in Denmark. The prevalence of diagnosed keratoconus in the Netherlands National Patient Register 1977–2015 was 44 per 100,000 persons [7].

In a recent study, it was observed that the prevalence of keratoconus in southern Spain was 30 per 100,000 [8].

The diagnosis of keratoconus is clinical. Therefore, it is established when a patient presents progressive loss of vision that is not corrected with glasses and is accompanied by biomicroscopic findings in the exploration.

Subclinical Keratoconus is defined as early stages of the disease, where visual acuity is usually preserved [5].

Throughout history, several classifications of clinical keratoconus have been used; the Amsler-Krumeich classification has been the most widely used. Alió-Shabayek modified it including coma-like corneal aberrations [9]. However, there is no adequate classification to determine the stage of this pathology at an early stage.

Corneal topography is a non-invasive diagnostic test that allows knowing the surface of the cornea. It was established that this is the best method of diagnosis in early keratoconus [10]. The Oculus Pentacam® system provides the anterior and posterior topographic, pachymetric and aberrometry maps.

The anterior corneal surface is the most critical refractive component of the eye, and its aberrations are very useful in the diagnosis of the corneal disease [10–14]. However, studies of aberrations of the posterior surface are discordant and inconclusive [11–16].

The study of corneal aberrations in incipient stages has allowed us to affirm that the anterior coma to 90° is the one that most discriminates them from healthy corneas [17]. Parameters as minimum corneal thickness, posterior coma [18], trefoil [19] and spherical aberration [16] would also have an influence.

It has been analyzed that corneal aberrations, especially the anterior coma to 90° and its influence in the visual quality of patients with keratoconus [18].

The study of the wavefront has great importance for the early diagnosis of keratoconus and the determination of variables that influence visual acuity. The main objective of this study is to establish a predictive model of early diagnosis in keratoconus with topographic variables obtained by Pentacam®.

## Methods

A cross-sectional study was carried out to analyses the topographic, pachymetric and aberrometry variables obtained by rotary Scheimpflug camera (Pentacam® type) from patients diagnosed with keratoconus, subclinical keratoconus and normal corneas in the Ophthalmology Service at the Torrecárdenas University Hospital (Almería, Spain) between February 2018 and February 2019. The data have been collected from the Pentacam® clinical database.

The sample size has been estimated with the Ene 3.0 calculator for the mean of a continuous variable (total corneal coma) in three pre-established strata. Thus, we based on the study by Prakash et al. [20] where values of total corneal coma aberration (μm) were obtained in normal patients (0.3 ± 0.1), subclinical (early) keratoconus (0.5 ± 0.3) and clinical keratoconus (2.1 ± 1.3). The reference population is all patients over or equal to 18 years old with keratoconus in the province of Almeria. After consulting the Institute of Statistics and Cartography of Andalusia, the total population census is 706,672 inhabitants, with those under 18 years of age (143,523 inhabitants). Therefore, the total estimated population is 563,149 inhabitants. The number of keratoconus in this population would be 168 cases, if we consider that its prevalence is 30/100,000, according to the recent study by Fernández-Barrientos et al. [8]. Estimated prevalence and clinical characteristics of keratoconus in the healthcare setting of the Hospital Costa del Sol, Spain. J Emmetropia 2014;5:15–21). To achieve a precision of 0.12 units in the estimation of a mean by means of a 95% bilateral confidence interval and assuming that the standard deviations of each stratum are those obtained in the previous study, it is necessary to include a total of 188 experimental units distributed among the 3 strata with proportions of 40% (*n* = 75), 20% (*n* = 38) and 40% (n = 75).

A total of 205 eyes of 205 patients (only one eye per patient) was distributed in 3 groups.

- Group 1: Healthy patients without corneal pathology,
- Group 2 Patients with early-stage keratoconus (ESKC). This group included patients with an eye with topographic signs of keratoconus and/or suspicious topographic findings under normal slit-lamp examination and keratoconus in the fellow eye as recently defined by Henriquez et al. [21].
- Group 3 Patients with keratoconus (KC). They must present at least one biomicroscopic alteration of the anterior segment (central thinning with Fleischer’s ring and Vogt’s striae) and the topography compatible with corneal ectasia. In patients with bilateral keratoconus, one of the eyes had been taken randomly.

The exclusion criteria were to have any systemic or ocular pathology and any ocular surgical intervention, including intrastromal rings and cross-linking.

A complete ophthalmological examination was performed in all cases.

Uncorrected visual acuity (UCVA) and best-corrected visual acuity (BCVA) were collected with Snellen’s chart (decimal scale). Objective refraction obtained by an autorefractometer (KR8900, Topcon, Japan) biomicroscopy (Carl Zeiss Meditec AG, Jena, Germany) and fundus were examined.

A corneal topographic analysis was performed on all patients by the same trained physician, under the same dark conditions and a pupil diameter of 6 mm. Patients with soft contact lenses didn’t wear them for three weeks, and the gas-permeable rigid lenses for at least five weeks before the test. The examination was performed with the rotary camera Scheimpflug (Pentacam® AXL, Oculus Optikgeräte, Wetzlar, Germany).

The following variables were collected:

Corneal topography of the anterior face: minor curvature (K1), major curvature (K2), mean curvature (Km), maximum curvature (KMAX), asphericity (Q), vertical asymmetry index (VAI); corneal topography of the posterior face: minor curvature (K1), major curvature (K2), mean curvature (Km) and asphericity (Q), central corneal thickness (CCT), minimum corneal thickness (MCT) with its coordinates (x,y) mean square root of total aberrations (Total RMS), mean square root of high order aberrations (HOA RMS), secondary corneal astigmatism to 0° (Z2^2^) and 45° (Z2^− 2^), anterior horizontal coma to 0°, posterior horizontal coma to 0°, total horizontal corneal coma to 0° (Z3^1^), anterior vertical coma to 90°, posterior vertical coma to 90°, total vertical corneal coma to 90° (Z3^− 1^), trefoil to 0° (Z3^− 3^), trefoil to 30° (Z3^3^), tetrafoil to 0° (Z4^4^), tetrafoil to 22.5° (Z4^− 4^) and spherical aberration (Z4^0^) and the Pentacam diagnostic indexes: BAD-D, IHR, PPI, ArtMax and IVA.

Statistical analysis was performed using the software for Windows SPSS (version 25.0, SPSS, Chicago, Illinois, USA) and R (version 3.5.1). A bivariate analysis was performed, previously checking the normality of the variables with the Kolmogorov Smirnoff test. The non-parametric Wilcoxon rank-sum test (Mann-Whitney test) was used for two samples and Kruskal Wallis test for more than two samples. For the binary logistic regression model, early keratoconus and normal groups were used as a dichotomous dependent variable, and stepwise method, direction forward-backwards and AIC criteria (Akaike information criteria) were used to include all possible predictor variables and to eliminate those that didn’t add value to the study (according to AIC criteria). The binary regression model is expressed in the form of an algorithm:

$$ {\displaystyle \begin{array}{l}\mathrm{Logit}\ \left(\mathrm{p}/1\hbox{-} \mathrm{p}\right)=\hbox{-} \mathrm{a}\hbox{-} {\mathrm{x}}_1{\upbeta}_1\hbox{-} {\mathrm{x}}_2{\upbeta}_2+{\mathrm{x}}_3{\upbeta}_3\\ {}\mathrm{Odds}\ \mathrm{Ratio}\ \left(\mathrm{OR}\right)={\mathrm{e}}^{\hbox{-} \mathrm{a}\hbox{-} \mathrm{x}1\upbeta 1\hbox{-} \mathrm{x}2\upbeta 2+\mathrm{x}3\upbeta 3}\\ {}\mathrm{a}=\mathrm{constant}\\ {}{\mathrm{x}}_{1,}{\mathrm{x}}_{2,}{\mathrm{x}}_{3=}\mathrm{coefficients}\ \mathrm{of}\ \mathrm{the}\ \mathrm{model}\\ {}{\upbeta}_{1,}{\upbeta}_{2,}{\upbeta}_{3=}\mathrm{Variables}\ \mathrm{of}\ \mathrm{the}\ \mathrm{model}\\ {}\mathrm{p}/1\hbox{-} \mathrm{p}=\mathrm{Odds}\ \mathrm{Ratio}\end{array}} $$

Once the coefficients have been calculated, the model is validated by evaluating the variance influence factors (VIF), which indicate that the correlation between the variables is low (less than 2). The Hosmer-Lemeshow test evaluated the fit of the model ROC curve calculated the AUC (Area under the curve) and the confusion matrix (actual vs predicted group) estimated the accuracy, precision, sensitivity and specificity of the diagnostic indices and discrimination functions performed on the validation set.

## Results

The study compared 205 eyes divided into three study groups, the distribution of which is shown in Table 1. There were no statistically significant differences in laterality or sex between the groups.

**Table 1:** Demographic characteristics

	Normal	ESKC	KC	ESKC Vs Controls	Controls Vs KC
				*p*-value*	*p*-value**
Patients n (%)	82 (39.8)	40 (19.4)	83 (40.3)		
Eye					
Right	41 (50.0)	19 (47.5)	54 (65.1)		0.078
Left	41 (50.0)	21 (52.5)	29 (34.9)		
Sex					
Male	36 (43.9)	23 (57.5)	40 (48.2)		0.369
Female	46 (56.1)	17 (42.5)	43 (51.8)		
Sphere* (D)	−0.36 (3.02) [−8; 4.50]	−1.06 (1.71) [−5.50; 3]	−3.71 (4.71) [− 16;6]	0.012	< 0.01
Cylinder* (D)	− 1.82 (2.15) [−6; 3.75]	−1.19 (0.99) [−2.50; 2.75]	−2.95(1.46) [− 6;1]	0.059	< 0.01
Spherical equivalent* (D)	−1.38(3.23) [− 10; 5.50]	−1.73(1.62) [− 5.50; 2.25]	−4.84(4.61) [− 18; 4.50]	0.251	< 0.01
BCVA* (decimal scale)	0.97(0.07) [0.7;1]	0.99(0.06) [0.7;1]	0.6(0.29) [0.05; 1]	0.219	< 0.01

*mean (sd) **p < 0.05

There were statistically significant differences between the three groups (*p* <  0.05, Kruskal-Wallis) for the sphere, cylinder, spherical equivalent and BCVA (decimal scale). Also, there were statistically significant differences between group 1 and 2 for the sphere (*p* = 0.012, U Mann-Whitney), (Table 1).

Means and standard deviations were calculated for the different variables. Those of more considerable clinical significance are presented in Table 2.

**Table 2:** Main Pentacam indices^a^ and bivariate analysis

	Controls	ESKC	KC	ESKC Vs Controls *p*-Value	KC Vs Controls p-Value
**Anterior surface topography**					
Km^b^	43.55(1.43)	43.37(1.55)	48.26(4.64)	0.616	
KMAX^b^	45.49(1.92)	45.91(1.97)	55.14(7.66)	0.285	< 0.01
IVA	0.16(0.08)	0.28(0.14)	0.79(0.51)	< 0.01	< 0.01
**Posterior surface topography**					
Km	−6.246(0.22)	−6.148(0.34)	−7.15(0.9)	0.067	< 0.01
Pachymetry					
CCT^c^	543.76(36.42)	515.20(27.59)	466.92(55.94)	< 0.01	< 0.01
MCT^c^	538.52(37.03)	503.67(26.62)	456.93(50.65)	< 0.01	< 0.01
**Corneal Aberrometry**^**d**^					
RMS HOA	0.52(0.23)	0.69(0.31)	1.74(1.02)	< 0.01	< 0.01
Ant Coma 90°	0.01(0.20)	− 0.49(0.43)	−2.06(1.51)	< 0.01	< 0.01
Post Coma 90°	−0.01(0.05)	0.11(0.10)	0.53(0.39)	< 0.01	< 0.01
Coma 90°	0.01(0.21)	−0.40(0.32)	−1.88(1.41)	< 0.01	< 0.01
Trefoil 0°	0.03(0.18)	0.08(0.22)	0.09(0.34)	0.396	< 0.01
Sph. Aberrat	0.20(0.14)	0.18(0.16)	−0.279(0.75)	0.204	< 0.01

^a^mean (sd) CCT = Central Corneal Thickness, MCT = Minimum corneal thickness, Sph Aberrat = Spherical Aberration, ^b^diopters ^c^μm ^d^RMS (μm)

### Early diagnosis of Keratoconus

The binary logistic model has been calculated using the forward-backwards method introducing variables and evaluating their statistical significance. Table 3 contains the *p*-values of the variables.

**Table 3:** Binary logistic regression model with all variables

	β	S.E.	Wald	df	Sig.	Exp(β)(OR)
Kmax	−0.11	0.18	0.39	1	0.53	0.9
Q	−1.1	1.81	0.37	1	0.54	0.33
MCT	−0.05	0.01	12.77	1	0.00*	0.95
Ant coma 0°	1.65	2.51	0.43	1	0.51	5.19
Post coma 0°	−14.65	10.81	1.84	1	0.18	0
Ant coma 90°	−4.13	2.15	3.68	1	0.06**	0.02
Post coma 90°	24.97	9.02	7.67	1	0.01*	69,920,300,645
Trefoil 0°	−1.56	1.79	0.75	1	0.39	0.21
Trefoil 30°	−0.81	1.59	0.26	1.00	0.61	0.44
Tetrafoil 0°	−0.02	2.22	0.00	1.00	0.99	0.98
Tetrafoil 22.5°	2.15	4.27	0.25	1.00	0.61	8.59
Sph Aberrat	−3.90	3.73	1.10	1.00	0.29	0.02
Constant	27.81	10.56	6.94	1.00	0.01	1,199,896,240,521.74

*p < 0.05, **p < 0.1. Dependent variable: Normal vs ESKC. MCT = Minimum corneal thickness, Sph Aberrat = Spherical Aberration

The initial criteria for the variable selection were *p*-value. After application of the AIC criteria, Table 4 analyzes the proposed model with the variables and their coefficients of the equation, including Odds Ratio (OR) (Exp(β)). The value of the coefficients is centred on the mean of each other for better understanding ((MCT mean 527 μm, Anterior coma 90° mean − 0.16 RMS (μm), Post coma 90° mean 0.03 RMS (μm)). Table 5 evaluates the matrix correlation among the variables. The elimination of the variable anterior coma to 90° (*p* = 0.11) decreased the calibration of the model. Although the correlation between anterior and posterior coma is 0.57 the variance inflation factor (VIF) is < 2 and the variable was kept within the model.

**Table 4:** Coefficients of the binary logistic regression model

Coefficients:	β	Exp(β)(OR)	Std. Error	z value	Pr(>|z|)	
(Intercept)	−1.89	0.15	0.59	− 3.19	0.00	*
Ant coma 90°_cent	−2.47	0.08	1.57	−1.57	0.11	
MCT527	−0.04	0.96	0.01	−3.46	0.00	*
Post coma 90°_cent	19.03	183,917,896	6.81	2.8	0.01	*

*p < 0.05, AIC = 79,10, Ant coma 90°_cent = Ant coma 90°-0.16, MCT527 = MCT-527, Post coma 90°_cent = Post coma 90°-0.03

**Table 5:** Matrix correlation among variables of the logistic regression model

	Constant	Post coma 90°	MCT	Ant coma 90°	VIF^a^
Constant	1	0.36	−0.99	0.10	
Post coma 90°	0.36	1	−0.36	0.57	1.75
MCT	−0.99	−0.36	1	−0.08	1.18
Ant coma 90°	0.10	0.57	−0.08	1	1.53

^a^VIF = Variance inflation factor

The proposed ESKC model expressed in the form of an algorithm is:

$$ {\displaystyle \begin{array}{l}\mathrm{Logit}\ \left(\mathrm{p}/1\hbox{-} \mathrm{p}\right)=\hbox{-} 1.89\hbox{-} 2.47{\upbeta}_1\hbox{-} 0.04{\upbeta}_2+19.03{\upbeta}_3\\ {}\ \mathrm{Odds}\ \mathrm{Ratio}\ \left(\mathrm{OR}\right)={\mathrm{e}}^{\hbox{-} 1.89\hbox{-} 2.47\upbeta 1\hbox{-} 0.04\upbeta 2+19.03\upbeta 3}\\ {}\mathrm{p}=\mathrm{early}\hbox{-} \mathrm{stage}\ \mathrm{keratoconus}\ \mathrm{p}\mathrm{robability}\\ {}{\upbeta}_1=\mathrm{Anterior}\ \mathrm{coma}\ 90 \mathrm{^0}\hbox{-} 0.16\ \mathrm{RMS}\ \left(\upmu \mathrm{m}\right)\\ {}{\upbeta}_2=\mathrm{MCT}\hbox{-} 527\ \upmu \mathrm{m}\\ {}{\upbeta}_3=\mathrm{Posterior}\ \mathrm{coma}\ 90 \mathrm{^0}\hbox{-} 0.03\ \mathrm{RMS}\ \left(\upmu \mathrm{m}\right)\end{array}} $$

where p is the probability of the early-stage keratoconus and (p/1-p) is the Odds Ratio (OR) that means the probability of ESKC divided by the probability of normal cases (1-p).

### The interpretation with the effect of each variable selected

The increased minimum corneal thickness (over 527 μm) and anterior coma to 90° with a positive sign (over − 0.16 RMS (μm)) decrease the probability of ESKC. Probability of ESKC is increased with posterior coma to 90° (over 0.03 RMS (μm)) with a positive sign and anterior coma to 90° with a negative sign (under − 0.16 RMS (μm)).

Table 5 evaluates the matrix correlation among the variables. The elimination of the variable anterior coma to 90° (*p* = 0.11) decreased the calibration of the model. Although the correlation between anterior and posterior coma is 0.574 the variance inflation factor (VIF) is < 2 and the variable was kept within the model.

Tables 6 and 7 describe the confusion matrix (actual vs predicted group) and the accuracy, precision, sensitivity and specificity of the diagnostic indices and discrimination functions performed on the validation set.

**Table 6:** Confusion matrix Normal vs ESKC

	Actual group	Total	Predicted group		AUC	Accuracy(%)	Precision(%)	Sensitivity(%)	Specificity(%)
			Normal	ESKC					
Proposed model	Normal	82	79	3	0.92	89.34	90.9	75	96.34
	ESKC	40	10	30					
BAD-D	Normal	82	74	8	0.93	85.25	78.95	75	90.24
	ESKC	40							
PPI	Normal	82	77	5	0.85	81.15	81.48	55	93.9
	ESKC	40							
IHD	Normal	82	75	7	0.82	77.87	74.07	50	91.46
	ESKC	40							
ArtMAX	Normal	82	75	7	0.88	83.61	79.41	67.5	91.46
	ESKC	40							
IVA	Normal	82	74	8	0.77	72.95	65.22	37.5	90.24
	ESKC	40	25	15					

BAD-D: Belin/Ambrósio Enhanced Ectasia Display. PPI-Avg: Mean Pachymetry Progression Index IHD: index of height decentration Art-MAX: The Ambrósio relational thickness maximum, IVA: vertical asymmetry index, MCT: minimum corneal thickness

**Table 7:** Confusion matrix Normal vs Keratoconus

	Actual group	Total	Predicted group		AUC	Accuracy(%)	Precision(%)	Sensitivity(%)	Specificity(%)
			Normal	KC					
Proposed model	Normal	82	81	1	0.99	98.18	98.78	97.59	98.78
	KC	83	2	81					
BAD-D	Normal	82	82	0	1	98.18	100	96.38	100
	KC	83	3	80					
PPI	Normal	82	80	2	0.98	95.15	97.46	92.77	97.56
	KC	83	6	77					
IHD	Normal	82	79	3	0.99	93.94	96.2	91.56	96.34
	KC	83	7	76					
ArtMAX	Normal	82	76	6	0.99	93.33	92.85	93.97	92.68
	KC	83	5	78					
IVA	Normal	82	77	5	0.98	91.52	93.67	89.15	93.9
	KC	83	9	74					

BAD-D: Belin/Ambrósio Enhanced Ectasia Display. PPI-Avg: Mean Pachymetry Progression Index IHD: index of height decentration Art-MAX: The Ambrósio relational thickness maximum, IVA: vertical asymmetry index, MCT: minimum corneal thickness

Table 8 compares the results obtained with the Pentacam neural network to validate the ESKC model.

**Table 8:** Comparative table with Pentacam neural network

Model [Study]	Sensitivity (%)	Specificity (%)	Accuracy (%)	Precision (%)
TKC [2]	58.00	99.70	79.00	99.62
BAD-D [2]	83.00	95.00	89.00	95.00
CMM-FFN [2]	90.00	94.35	93.67	94.27
CAD [2]	97.78	95.56	96.56	95.65
SVM [1], *(Sirius)*	92.00	97.70	97.30	78.80
PIRSS [4]	92.00	99.10	98.00	
ESKC MODEL^a^	97.59	98.78	98.18	98.78

^a^own results

Finally, a comparative table of the results obtained in this work with the results obtained by other authors (Table 9). This table facilitates the discussion of the results obtained in this research.

**Table 9:** Comparative table of studies evaluating Pentacam topographic, pachymetric and aberrometry parameters in detecting subclinical (early) keratoconus (confusion matrix)

Study	Sensitivity (%)	Specificity (%)	AUC
BAD-D^a^ [3]			
Ferreira-Mendes et al. [22]	68.40	84.60	0.839
Huseynli et al. [9]	95.50	73.70	0.904
Hashemi et al. [23]	81.10	73.20	0.860
Shetty et al. [24]	83.80	86.00	0.887
Ruiseñor Vázquez et al. [25]	89.20	82.30	0.930
Ambrósio et al. [26]	93.60	94.60	0.975
Steinberg et al. [27]	65.80	65.80	0.712
Muftuoglu et al. [28]	60.00	90.00	0.834
Castro-Luna et al.^b^	75.00	96.34	0.930
PPI-Avg^a^ [3]			
Cui et al. [29]	94.70	89.70	0.957
Huseynli et al. [9]	93.30	47.40	0.834
Shetty et al. [24]	83.80	74.40	0.883
Ruiseñor Vázquez et al. [25]	78.40	82.80	0.860
Muftuoglu et al. [28]	77.00	65.00	0.806
Steinberg et al. [27]	62.30	64.30	0.669
Uçakhan et al. [30]	81.80	77.80	0.842
Castro-Luna et al.^b^	75.00	90.24	0.850
IHD^a^ [3]			
Kovács et al. [31]	80.00	75.00	0.880
Bae et al. [32]	71.40	85.30	0.748
Huseynli et al. [9]	82.30	65.00	0.782
Shetty et al. [24]	43.20	67.40	0.627
Uçakhan et al. [30]	75.00	60.30	0.703
Castro-Luna et al.^b^	50.00	91.46	0.820
Art-MAX^a^ [3]			
Kovács et al. [31]	84.00	54.00	0.740
Shetty et al. [24]	86.50	69.80	0.850
Muftuoglu et al. [28]	67.00	71.00	0.722
Ruiseñor Vázquez et al. [25]	90.50	86.50	0.930
Ambrósio et al. [26]	85.10	93.10	0.959
Steinberg et al. [27]	30.80	30.60	0.272
Castro-Luna et al.^b^	67.50	91.46	0.880
IVA^a^ [3]			
Bae et al. [32]	71.40	61.80	0.733
Huseynli et al. [9]	92.10	52.50	0.844
Hashemi et al. [23]	82.30	73.20	0.860
Shetty et al. [24]	10.80	95.30	0.609
Uçakhan et al. [30]	86.40	61.90	0.768
Castro-Luna et al.^b^	37.50	90.24	0.770
MCT^a^ [3]			
Kovács et al. [31]	64.00	66.00	0.670
Xu et al. [33]	92.00	47.00	0.695
Cui et al. [29]	84.20	100.00	0.914
Reddy et al. [34]	70.00	80.00	0.790
Muftuoglu et al. [28]	64.00	58.00	0.652
Uçakhan et al. [30]	88.90	61.40	0.805
Steinberg et al. [27]	36.30	35.70	0.323
Castro-Luna et al.^b^	45.00	87.80	0.780
Anterior coma to 90° [5]			
Heidari et al. [35]	75.00	100.00	0.857
Castro-Luna et al.^b^	67.50	93.90	0.86
Posterior coma to 90° [5]			
Heidari et al. [35]	75.00	78.6	0.813
Castro-Luna et al.^b^	62.50	93.90	0.85

^a^BAD-D: Belin/Ambrósio Enhanced Ectasia Display. PPI-Avg: Mean Pachymetry Progression Index IHD: index of height decentration Art-MAX: The Ambrósio relational thickness maximum, IVA: vertical asymmetry index, MCT: minimum corneal thickness. ^b^ own results

## Discussion

Detection of ESKC has always been a challenge for ophthalmologists, especially when there are no clinical signs or symptoms in the patient.

The rotary camera Scheimpflug (Pentacam®) topography is usually used to diagnose keratoconus in daily clinical practice [15, 16, 20, 23, 33, 36–40]. The topographic parameters of clinical keratoconus are recognizable. However, it is not easy to diagnose subclinical keratoconus based on topographic variables. This study calculates a diagnostic model based on the aberrometry data of the anterior and posterior corneal surface provided by the Pentacam.

The selection of the sample was made that there were no differences between the age groups [14–17, 19–21, 34, 39, 41, 42], sex [16, 20, 37, 42], and eye [20, 37]. This is an advantage when interpreting results that aren’t biased by age and sex. As reported by Koçamis et al. [37], there are significant differences for age between keratoconus (26.19 ± 7.90) and healthy (30.88 ± 7.57).

Pupillary dilatation is another parameter that can modify the aberrometry results [43]. In this study, it was prefixed in 6 mm. In previous studies [8, 12–14, 21, 23, 34, 36, 41] like Hondur et al. [43] established it in 5 mm.

Many studies have been made between healthy patients with Keratoconus [10–12, 14–16, 20, 23, 36–40, 43] or healthy patients with ESKC [17–21, 23, 33, 34, 37, 38, 41, 42, 44, 45]. The purpose in most of them was to analyze the topographic parameters to find differences between a healthy patient and an incipient corneal ectasia without symptoms. However in all of them, many different classification methods have been used: Amsler-Krumeich [39, 43, 44], Alió and Shabayek [15, 36], KISA % index [20] or KSS [42]. All this methodological variability leads to an outstanding selection and classification bias when making comparisons between studies.

If we analyze the refractive parameters of this study, statistically significant differences were obtained between the three groups analyzed for the sphere, the cylinder and the spherical equivalent (*p* <  0.05, Kruskal-Wallis), as in other studies [33, 34]. However, when comparing normal corneas with ESKC, we obtained statistically significant differences only for the sphere (*p* = 0.012, U Mann-Whitney). Saad and Gatinel [19] obtained that the mean of the sphere was significantly higher in their normal group than in their ESKC group (*p* < 0.001). Reddy et al. [34] observed statistically significant differences for the cylinder (p < 0.001) not for the sphere (*p* = 0.08). However, Naderan et al. [42] didn’t find statistically significant differences for sphere (*p* = 0.136) or cylinder (*p* = 0.108). In this study, there are statistically significant differences between the BCVA of the three groups, but it wasn’t differences between normal corneas and ESKC. These values are consistent with previous studies [10, 20, 33, 36, 37, 43, 45]. When we analyzed a bivariate analysis between normal corneas and ESKC, statistically significant differences were only obtained for variables of vertical asymmetry, total coma to 90° and corneal thickness (*p* < 0.05). Bührenet al [17], found that the anterior coma to 90° would be the most useful parameter to differentiate normal corneas from ESKC. Other parameters such as the posterior coma to 90° and the minimum corneal thickness didn’t exceed the value of the anterior surface for this author. In this study, when the total corneal coma to 90° was analyzed in absolute value, we found that it was higher in ESKC (|-0.404| ± 0.319) than in normal (0.0123 ± 0.209), but lower than in keratoconus (|-1.877| ± 1.413). This value indicates that the parameter total corneal coma to 90° had increased with the natural history of the disease [19]. The negative sign of the corneal coma to 90° refers to the lower decentration of the cone in the y-axis [19]. More recently, Naderan et al. [42] and Xu et al. [33] indicated the importance of posterior surface aberrations to differentiate normal from ESKC corneas. In the first study, they obtained that the values for posterior coma to 90° of the healthy group were 0.032 ± 0.363 and for the ESKC group were 0.193 ± 0.264 with statistically significant differences between groups (*p* = 0.003, U Mann-Whitney). In this database, the posterior coma to 90° for normal corneas were − 0.008 ± 0.049 and for ESKC were 0.112 ± 0.103, (*p* < 0.05, U Mann-Whitney). The relationship between coma-like aberrations of the anterior surface and the degree of manifest keratoconus is well known [10, 14, 23, 37, 39, 40, 43]. Piñero et al. [15] were the first to attempt to characterize the posterior corneal surface and its aberrations in patients with normal corneas and keratoconus, finding results that were not concordant by the optical theory of the corneal surface. Piñero et al. have obtained values of anterior coma to 90° of 0.001 ± 0.225 and posterior coma to 90° of 0.319 ± 0.372 from the healthy patients. In keratoconus were − 1.754 ± 0.976 and − 3.692 ± 1.81 respectively.

If we analyze the results of this study, in healthy patients the anterior coma to 90° was 0.01 ± 0.20, and posterior coma to 90° was − 0.01 ± 0.05 (the same mean but opposite sign), and in keratoconus, we obtained − 2.06 ± 1.51 and 0.53 ± 0.38 respectively. In this case, the anterior coma to 90°, in absolute value, were higher than the posterior ones. In subclinical keratoconus, the anterior coma to 90° was − 0.49 ± 0.43, and the posterior coma to 90° was 0.11 ± 0.10. Comparing the results, we observed that both anterior and posterior coma at 90° increase with the appearance of the corneal alterations of keratoconus from early stages but with opposite signs, while the anterior coma to 90° becomes negative, the posterior coma to 90° becomes positive.

Attempted to other parameters, several studies like Buhrenet al [17] observed that the MCT was the most discriminating parameter between normal corneas and ESKC. However, they concluded that the posterior surface was not discriminate as to the anterior surface, and this surface was not sufficient for the diagnosis of the subclinical keratoconus. Otherway Safarzadeh et al. [44] reflected that minimum corneal thickness and posterior corneal elevation would be the best parameters for differentiating suspicious keratoconus from healthy eyes in concordance with our results.

The main purpose of this study is to calculate a binary logistic model to predictive the early stage keratoconus. Other authors [19, 20, 33] have established binary logistic models for keratoconus diagnosis but not for early-stage keratoconus. The results analyze the probability of ESKC using three variables: MCT, anterior coma to 90° and posterior to 90°. The validation of this model, with the Hosmer Lemeshow test and the AUC, suggest a good calibration in 92% of cases. Observing the confusion matrix constructed with the actual versus predicted values, the discrimination of the early keratoconus in the ESKC model has a specificity (96.34), precision (90.9) and accuracy(89.34) superior to the diagnostic indices of Pentacam. In the discrimination of normals and keratoconus (table), the sensitivity of the ESKC model (97.59) is higher than the diagnostic indices of Pentacam. According to the comparative table with the Pentacam neural network designed for keratoconus detection, the ESKC model has a sensitivity (97.59) similar to CAD (97.78), but the specificity (98.78), accuracy (98.18) and precision (98.78) are superior. Finally, Table 9 shows a comparison of the results of the diagnostic indices of Pentacam according to the different authors. There are heterogeneous results, probably for the unclear criteria to define the early-stage keratoconus.

The main limitation of this study is the sample size of early-stage keratoconus, whose number is lower than keratoconus. Another limitation is the already mentioned diagnostic criteria of the early-stage keratoconus. There is a considerable disparity in the results of the different authors, probably for the above reason especially in no recent publications.

## Conclusions

The most important aberrometry parameters in the diagnosis of keratoconus are those related to vertical asymmetries: specifically the anterior coma to 90° and the posterior coma to 90°, in addition to the minimum corneal thickness. In the case of the diagnosis of early-stage keratoconus, the main parameter is the increase with a positive sign of the posterior coma to 90°.

## Data Availability

The datasets generated and/or analysed during the current study are available in the KERATOCONUS repository, Castro de Luna, Gracia (2020), “KERATOCONUS WITH PENTACAM INDICES”, Mendeley Data, V3, doi: 10.17632/fcb47rngbd.3

## Abbreviations

ESKC: Early stage Keratoconus
MCT: Minimum Corneal Thickness
UCVA: Uncorrected Visual Acuity
BCVA: Best Corrected Visual Acuity
Km: Mean curvature (Km),
Kmax: maximum curvature (KMAX),
Q: asphericity
VAI: vertical asymmetry index
Total RMS: mean square root of total aberrations
HOA RMS: mean square root of high order aberrations
AUC: Area Under Curve
ROC curve: Receiver Operating Characteristics curve

## References

[1] Mas Tur V, MacGregor C, Jayaswal R, O’Brart D, Maycock N (2017). A review of keratoconus: diagnosis, pathophysiology, and genetics. SurvOphthalmol..

[2] Moussa S, Grabner G, Ruckhofer J, Dietrich M, Reitsamer H (2017). Genetics in Keratoconus – What is New?. Open Ophthalmol J.

[3] Naderan M, Shoar S, Rezagholizadeh F, Zolfaghari M, Naderan M (2015). Characteristics and associations of keratoconus patients. Contact Lens Anterior Eye.

[4] Gordon-Shaag A, Millodot M, Shneor E, Liu Y. The Genetic and Environmental Factors for Keratoconus. Biomed Res Int. 2015;2015:795738. 10.1155/2015/795738. 10.1155/2015/795738 PMC4449900 26075261

[5] Romero-Jiménez M, Santodomingo-Rubido J, Wolffsohn JS (2010). Keratoconus: a review. Contact Lens Anterior Eye..

[6] Bak-Nielsen S, Ramlau-Hansen CH, Ivarsen A, Plana-Ripoll O (2019). Hjortdal incidence and prevalence of keratoconus in Denmark - an update. J Acta Ophthalmol.

[7] Godefrooij DA, de Wit GA, Uiterwaal CS, Imhof SM, Wisse RP (2017). Age-specific incidence and prevalence of Keratoconus: a Nationwide registration study. Am J Ophthalmol.

[8] Fernández-Barrientos Y, Gismero-Moreno S, Lorenzo-Soto M (2014). Estimated prevalence and clinical characteristics of keratoconus in the healthcare setting of the hospital Costa del Sol, Spain. J Emmetropia.

[9] Huseynli S, Abdulaliyeva F (2018). Evaluation of Scheimpflug tomography parameters in subclinical Keratoconus, clinical Keratoconus and Normal Caucasian eyes. TürkOftalmolDerg..

[10] Alió JL, Shabayek MH (2006). Corneal higher-order aberrations: a method to grade keratoconus. J Refract Surg.

[11] Gomes JAP, Tan D, Rapuano CJ, Belin MW, Ambrósio R, Guell JL, Malecaze F, Nishida K, Sangwan VS (2015). Global consensus on Keratoconus and Ectatic diseases. Cornea..

[12] Mounir A, El Saman IS, Anbar M (2019). The correlation between corneal topographic indices and corneal high order aberrations in Keratoconus. Med Hypothesis Discov Innov Ophthalmol.

[13] Oliveira CM, Ferreira A, Franco S (2012). Wavefront analysis and Zernike polynomial decomposition for evaluation of corneal optical quality. J Cataract Refract Surg.

[14] Maeda N, Fujikado T, Kuroda T, Mihashi T, Hirohara Y, Nishida K, Watanabe H, Tano Y (2002). Wavefront aberrations measured with a Hartmann-shack sensor in patients with keratoconus. Ophthalmology..

[15] Piñero DP, Alió JL, Alesón A, Escaf M, Miranda M (2009). Pentacam posterior and anterior corneal aberrations in normal and keratoconic eyes. Clin Exp Optom.

[16] Nakagawa T, Maeda N, Kosaki R, Hori Y, Inoue T, Saika M, Mihashi T, Fujikado T, Tano Y (2009). Higher-order aberrations due to the posterior corneal surface in patients with keratoconus. Investig Ophthalmol Vis Sci.

[17] Bühren J, Kook D, Yoon G, Kohnen T (2010). Detection of subclinical keratoconus by using corneal anterior and posterior surface aberrations and spatial thickness profiles. Investig Ophthalmol Vis Sci.

[18] Alió JL, Piñero DP, Alesón A, Teus MA, Barraquer RI, Murta J, Maldonado MJ, Castro de Luna G, Gutiérrez R, Villa C (2011). Keratoconus-integrated characterization considering anterior corneal aberrations, internal astigmatism, and corneal biomechanics. J Cataract Refract Surg.

[19] Saad A, Gatinel D (2012). Evaluation of total and corneal wavefront high order aberrations for the detection of forme fruste keratoconus. Investig Ophthalmol Vis Sci.

[20] Prakash G, Suhail M, Srivastava D (2016). Predictive analysis between topographic, Pachymetric and Wavefront parameters in Keratoconus, suspects and Normal eyes: creating unified equations to evaluate Keratoconus. Curr Eye Res.

[21] Henriquez MA, Hadid M, Izquierdo L (2020). A systematic review of subclinical Keratoconus and Forme Fruste Keratoconus. J Refract Surg.

[22] Ferreira-Mendes J, Lopes BT, Faria-Correia F, Salomao MQ, Rodrigues-Barros S, Ambrosio R (2019). Enhanced Ectasia detection using corneal tomography and biomechanics. Am J Ophthalmol.

[23] Hashemi H, Beiranvand A, Yekta A, Maleki A, Yazdani N, Khabazkhoob M (2016). Pentacam top indices for diagnosing subclinical and definite keratoconus. J CurrOphthalmol.

[24] Shetty R, Rao H, Khamar P, Sainani K, Vunnava K, Jayadev C (2017). Keratoconus screening indices and their diagnostic ability to distinguish Normal from Ectatic corneas. Am J Ophthalmol.

[25] Ruisenor Vazquez PR, Galletti JD, Minguez N, Delrivo M, Fuentes Bonthoux F, Pfortner T (2014). Pentacam Scheimpflug tomography findings in topographically normal patients and subclinical keratoconus cases. Am J Ophthalmol.

[26] Ambrosio R, Valbon BF, Faria-Correia F, Ramos I, Luz A (2013). Scheimpflug imaging for laser refractive surgery. Curr Opin Ophthalmol.

[27] Steinberg J, Aubke-Schultz S, Frings A, Hulle J, Druchkiv V, Richard G (2015). Correlation of the KISA% index and Scheimpflug tomography in 'normal', 'subclinical', 'keratoconus- suspect' and 'clinically manifest' keratoconus eyes. Acta Ophthalmol.

[28] Muftuoglu O, Ayar O, Hurmeric V, Orucoglu F, Kilic I (2015). Comparison of multimetric D index with keratometric, pachymetric, and posterior elevation parameters in diagnosing subclinical keratoconus in fellow eyes of asymmetric keratoconus patients. J Cataract Refract Surg.

[29] Cui J, Zhang X, Hu Q, Zhou WY, Yang F (2016). Evaluation of corneal thickness and volume parameters of subclinical Keratoconus using a Pentacam Scheimflug system. Curr Eye Res.

[30] Ucakhan OO, Cetinkor V, Ozkan M, Kanpolat A (2011). Evaluation of Scheimpflug imaging parameters in subclinical keratoconus, keratoconus, and normal eyes. J Cataract Refract Surg.

[31] Kovacs I, Mihaltz K, Kranitz K, Juhasz E, Takacs A, Dienes L (2016). Accuracy of machine learning classifiers using bilateral data from a Scheimpflug camera for identifying eyes with preclinical signs of keratoconus. J Cataract Refract Surg.

[32] Bae GH, Kim JR, Kim CH, Lim DH, Chung ES, Chung TY (2014). Corneal topographic and tomographic analysis of fellow eyes in unilateral keratoconus patients using Pentacam. Am J Ophthalmol.

[33] Xu Z, Li W, Jiang J, Zhuang X, Chen W, Peng M, Wang J, Lu F, Shen M, Wang Y (2017). Characteristic of entire corneal topography and tomography for the detection of sub-clinical keratoconus with Zernike polynomials using Pentacam. Sci Rep.

[34] Reddy JC, Rapuano CJ, Cater JR, Suri K, Nagra PK, Hammersmith KM (2014). Comparative evaluation of dual Scheimpflug imaging parameters in keratoconus, early keratoconus, and normal eyes. J Cataract Refract Surg.

[35] Heidari Z, Mohammadpour M, Hashemi H, et al. Early diagnosis of subclinical keratoconus by wavefront parameters using Scheimpflug, Placido and Hartmann–Shack based devices. Int Ophthalmol. 2020. 10.1007/s10792-020-01334-3. 10.1007/s10792-020-01334-3 32219617

[36] Bernal Reyes N, Arias Díaz A, Camacho Rangel LE (2015). Aberraciones corneales anteriores y posteriores medidas mediante imágenes de Scheimpflug en el queratocono en niños. RevMexOftalmol..

[37] Kocamış Sİ, Çakmak HB, Çağıl N, Toklu Y (2016). Investigation of the efficacy of the cone location and magnitude index in the diagnosis of Keratoconus. Semin Ophthalmol.

[38] Bayraktar Bilen N, Hepsen IF, Arce CG. Correlation between visual function and refractive, topographic, pachymetric and aberrometric data in eyes with keratoconus. Int J Ophthalmol. 2016;9(8):1127–33. 10.18240/ijo.2016.08.07. 10.18240/ijo.2016.08.07 PMC4990576 27588266

[39] Colak HN, Kantarci FA, Yildirim A, Tatar MG, Goker H, Uslu H, Gurler B (2016). Comparison of corneal topographic measurements and high order aberrations in keratoconus and normal eyes. Contact Lens Anterior Eye..

[40] Delgado S, Velazco J, Delgado Pelayo RM, Ruiz-Quintero N (2016). Correlación de aberraciones de alto orden en la cara anterior de la córnea y el grado de queratocono medidas con cámara de Scheimpflug. Arch Soc Esp Oftalmol.

[41] Bruce AS, Catania LJ (2014). Clinical applications of wavefront refraction. Optom Vis Sci.

[42] Naderan M, Jahanrad A, Farjadnia M (2018). Ocular, corneal, and internal aberrations in eyes with keratoconus, forme fruste keratoconus, and healthy eyes. Int Ophthalmol.

[43] Hondur G, Cagil N, Sarac O, Ozcan ME, Kosekahya P (2017). Pupillary offset in Keratoconus and its relationship with clinical and topographical features. Curr Eye Res.

[44] Safarzadeh M, Nasiri N (2016). Anterior segment characteristics in normal and keratoconus eyes evaluated with a combined Scheimpflug/Placido corneal imaging device. J Curr Ophthalmol.

[45] Aksoy S, Akkaya S, Özkurt Y, Kurna S, Açikalin B, Şengör T (2018). Topography and higher order corneal aberrations of the fellow eye in unilateral Keratoconus. Turkish J Ophthalmol.

